# Elevated Expression of *SLC6A4* Encoding the Serotonin Transporter (SERT) in Gilles de la Tourette Syndrome

**DOI:** 10.3390/genes12010086

**Published:** 2021-01-12

**Authors:** Mathis Hildonen, Amanda M. Levy, Christina Dahl, Victoria A. Bjerregaard, Lisbeth Birk Møller, Per Guldberg, Nanette M. Debes, Zeynep Tümer

**Affiliations:** 1 Kennedy Center, Department of Clinical Genetics, Copenhagen University Hospital, Rigshospitalet, 2600 Glostrup, Denmark; mathis.hildonen@regionh.dk (M.H.); marie.amanda.bust.levy@regionh.dk (A.M.L.); victoria.bjerregaard@regionh.dk (V.A.B.); Lisbeth.Birk.Moeller@regionh.dk (L.B.M.); 2 Danish Cancer Society Research Center, 2100 Copenhagen, Denmark; chd@cancer.dk (C.D.); perg@cancer.dk (P.G.); 3 Institute for Nature, Systems and Models, Roskilde University Center, 4000 Roskilde, Denmark; 4 Department of Cancer and Inflammation Research, Institute for Molecular Medicine, University of Southern Denmark, 5000 Odense, Denmark; 5 Tourette Clinics, Department of Paediatrics, Copenhagen University Hospital, 2730 Herlev, Denmark; nanette.marinette.monique.debes@regionh.dk; 6 Deparment of Clinical Medicine, Faculty of Health and Medical Sciences, University of Copenhagen, 2020 Copenhagen, Denmark

**Keywords:** SERT, *SLC6A4*, 5-HTT, Gilles de la Tourette syndrome, GTS, OCD, obsessive compulsive disorder, methylation, expression, serotonin

## Abstract

Gilles de la Tourette syndrome (GTS) is a complex neurodevelopmental disorder characterized by motor and vocal tics. Most of the GTS individuals have comorbid diagnoses, of which obsessive-compulsive disorder (OCD) and attention deficit-hyperactivity disorder (ADHD) are the most common. Several neurotransmitter systems have been implicated in disease pathogenesis, and amongst these, the dopaminergic and the serotonergic pathways are the most widely studied. In this study, we aimed to investigate whether the serotonin transporter (SERT) gene (*SLC6A4*) was differentially expressed among GTS individuals compared to healthy controls, and whether DNA variants (the SERT-linked polymorphic region 5-HTTLPR, together with the associated rs25531 and rs25532 variants, and the rare Ile425Val variant) or promoter methylation of *SLC6A4* were associated with gene expression levels or with the presence of OCD as comorbidity. We observed that *SLC6A4* expression is upregulated in GTS individuals compared to controls. Although no specific genotype, allele or haplotype was overrepresented in GTS individuals compared to controls, we observed that the L_AC_/L_AC_ genotype of the 5-HTTLPR/rs25531/rs25532 three-locus haplotype was associated with higher *SLC6A4* mRNA expression levels in GTS individuals, but not in the control group.

## 1. Introduction

Gilles de la Tourette syndrome (GTS) is a childhood-onset neurodevelopmental disorder characterized by at least one vocal and multiple motor tics, which begin before age 18 years and persist at least 1 year. Average age of onset is between 3 and 9 years with a male to female ratio of around 3:1 [1]. Comorbid conditions including obsessive-compulsive disorder (OCD), attention deficit-hyperactivity disorder (ADHD) and autism spectrum disorder (ASD) are present in more than 70% of the GTS individuals [1,2].

GTS is a complex disorder, with a largely unknown aetiology: several environmental factors are thought to interact with multiple genes in yet undiscovered ways. GTS has a high heritability estimate (>0.5) [3,4], but identification of susceptibility genes has been challenging likely due to the complex and heterogeneous genetic architecture, wherein common and rare variants in various genes and biological pathways are involved [5,6,7,8]. 

Neuroimaging and neurophysiology studies suggest that GTS is associated with altered synaptic neurotransmission systems involving dopamine, serotonin, inhibitory neurotransmitter γ-aminobutyric acid (GABA) and excitatory neurotransmitter glutamate in the cortico-striato-thalamo-cortical circuits [9,10,11]. Furthermore, candidate gene studies suggest involvement of dopaminergic [12,13,14,15], serotonergic [16,17], glutamatergic [18] and histaminergic [19] pathways in GTS pathogenesis. Dopamine neurotransmission is extensively studied in GTS pathology and an alteration of the tonic-phasic dopamine release is considered as a hallmark leading to the designation of the “dopaminergic hypothesis” [20,21]. The connection between serotonin neurotransmission and GTS has, however, been characterized to a lesser extent. As early as 1990, Comings reported decreased serotonin/platelet ratio in a large cohort of GTS individuals and family members [22], and a range of drugs with high affinity for serotonin receptors, mainly atypical antipsychotics, have been used to relieve tics [23]. However, even though the dysfunction of the serotonergic system is thought to be a primary cause in OCD [24], the extent of its involvement in GTS is yet unknown. Serotonin (5-hydroxytryptamine, 5-HT) receptors have been found to both facilitate and inhibit dopamine activity [25,26,27,28]. The serotonin transporter (5-HTT, SERT), which regulates serotonergic neurotransmission by retrieving serotonin from the synaptic cleft back to the presynaptic neuron, is capable of dopamine uptake, meaning that it also functions as a dopamine transporter [29]. Furthermore, SERT knockout rats have reduced expression of proteins essential for the glutamatergic synapses [30]. SERT binding potential (BP) has been investigated in GTS [31,32,33,34] and reduced SERT BP was observed in both GTS-only and GTS+OCD individuals [32,33] and negatively correlated with tic severity [31]. However, in a recent study, increased SERT BP was observed in GTS+OCD, but not in GTS-only or OCD-only individuals [34], and the differences in methodologies were suggested as an explanation [34]. The serotonin system may thus be involved in GTS pathology directly and/or indirectly through regulation of other neurotransmitter systems, especially the dopaminergic system. 

SERT is encoded by *SLC6A4*, which has been implicated in GTS aetiology by several studies: higher blood *SLC6A4* mRNA expression levels were found to correlate with tic severity in GTS [35], elevated *SLC6A4* expression was found in the striatum of rat models of GTS [36] and the rare *SLC6A4* gain-of-function (GOF) variant Ile425Val known to modulate SERT activity was reported to have a higher prevalence in GTS individuals compared to controls [17,37]. The SERT-linked polymorphic region (5-HTTLPR) in the promoter region immediately upstream to *SLC6A4* has been implicated in both OCD [38,39,40] and GTS aetiology [17]. The 5-HTTLPR polymorphic region is a 43 bp repeat with two common alleles, a long (L) and a short (S) allele. The L allele has been associated with higher *SLC6A4* mRNA expression in blood leading to increased SERT mediated serotonin clearance, and ultimately resulting in reduced serotonergic neurotransmission [41]. *SLC6A4* expression is further modulated by two 5-HTTLPR-adjacent single nucleotide polymorphisms (SNPs) rs25531 (A>G) and rs25532 (C>T). Initially, the 5-HTTLPR/rs25531 L_G_ allele was found to mimic the 5-HTTLPR S allele regarding expression levels, and only the L_A_ allele was found to have higher *SLC6A4* expression [39]. Later, when Wendland et al. investigated the variants of the *SLC6A4* promoter region including rs25532, and their effect on mRNA levels, they identified the three-locus haplotype L_AC_ (5-HTTLPR/rs25531/rs25532) as the highest expressing haplotype [38]. At the same time, they reported the L_AC_ haplotype to be overrepresented in OCD individuals compared to controls, and the same haplotype was later found to be more prevalent in GTS individuals compared to controls [17]. 

Methylation of the *SLC6A4* promoter region has been previously investigated in the peripheral blood of individuals with major depressive disorder [42], children with childhood physical aggression [43] and ADHD [44] and in the saliva of paediatric OCD [45]. In general, increased methylation was observed in affected individuals compared to controls, and hypermethylation of two CpG-sites was also correlated with increased *SLC6A4* mRNA expression levels in affected individuals [42]. Furthermore, methylation of *SLC6A4* in peripheral blood was correlated with in vivo human brain serotonin synthesis [43]. So far, there are not any studies investigating the methylation of the *SLC6A4* promoter region in GTS individuals, but elevated blood methylation levels of the dopamine D2 receptor gene (*DRD2*) was correlated with tic severity, while DNA methylation of the dopamine transporter (DAT) gene (*SLC6A3*) was lower in more severely affected individuals [46]. 

As associations between *SLC6A4* methylation, expression and gene variants have not been studied in GTS previously, we investigated whether *SLC6A4* was differentially expressed in GTS individuals with or without OCD compared to healthy controls and assessed whether gene variants or promoter methylation of *SLC6A4* were associated with gene expression levels. 

## 2. Materials and Methods 

### 2.1. GTS and Control Cohort 

In this study, we included only male individuals to exclude sex-specific methylation differences, as *SLC6A4* promoter region methylation has been shown to be higher in females [47] and GTS is more common in males [1]. The affected individuals are referred to as GTS individuals regardless of the presence of comorbid OCD unless otherwise noted (i.e., GTS-only or GTS+OCD). The GTS cohort comprised 72 male individuals (aged 16.1 ± 4.0) of whom 50 had only GTS (GTS-only), while 22 had GTS and OCD (GTS+OCD). RNA was available from 57 GTS individuals (43 GTS-only and 14 GTS+OCD). GTS individuals were recruited through the Herlev Tourette Clinic (Denmark) and the GTS diagnosis was established by an experienced neuropediatrician using DSM-IV-TR criteria (DSM-IV-TR, 2000) and validated clinical instruments were used to assess the presence of comorbidities as described previously [48]. The study was approved by the Danish Institutional Review Board (2011 H-2-2010-144). Control material comprised DNA from 87 anonymized male individuals (aged 17.7 ± 7.1), and RNA was available from 36 of them. The GTS and control cohort are summarized in Supplementary Table S1.

### 2.2. Genotyping

DNA was extracted from peripheral blood following standard procedures. Genotyping of 5-HTTLPR, rs25531, rs25532 (Supplementary Figure S1), and Ile425Val variants in *SLC6A4* were carried out with PCR followed by Sanger sequencing. PCR was carried out using the HotStarTaq^®^ DNA Polymerase kit (Qiagen, Hilden, Germany) with a no-template-control (NTC) included in each run. PCR products were purified using the MultiScreen^®^ PCR_µ96_ Plate (Millipore, Burlington, MA, USA) according to the manufacturer’s instructions and run on a 2% agarose gel (Sigma Aldrich, St. Louis, MO, USA). The long (L allele) and the short (S allele) fragments were excised from the gel and Sanger sequenced using the BigDye™ Terminator v3.1 Cycle Sequencing kit and analysed on an ABI 3730 DNA analyser (Applied Biosystems, Foster City, CA, USA). Only individuals homozygous for either the S or the L allele were sequenced for rs25531 and rs25532 determination. PCR and Sanger sequencing primers and conditions are listed in Supplementary Tables S2 and S3. 

### 2.3. Expression Analysis 

RNA was extracted from peripheral blood following standard procedures. mRNA expression levels of *SLC6A4* were quantified using reverse-transcription quantitative PCR (RT-qPCR). For this, 1 µg of total RNA was used for cDNA synthesis using the High-Capacity cDNA Reverse Transcription kit (Applied Biosystems) according to manufacturer’s instructions, with minor modifications (Supplementary Table S4). qPCR was carried out using TaqMan probes against either *SLC6A4* (#Hs00169010_m1; Applied Biosystems) or *GUSB* (#Hs00939627_m1; Applied Biosystems). All samples were amplified in triplicates on a 7500 Fast Real-Time PCR system (Applied Biosystems). The relative standard curve method was used for calculation and *SLC6A4* mRNA expression levels were normalized to *GUSB* mRNA levels. qPCR conditions are shown in Supplementary Table S5.

### 2.4. Methylation Analysis

Bisulphite pyrosequencing was used to quantify the degree of DNA methylation at eight CpG-sites within the 799 bp CpG-island at the promoter region of *SLC6A4* (chromosome position chr17: 28562388–28563186, GRCh37/hg19) (Supplementary Figure S2). DNA (200 ng) was bisulphite converted using an EZ DNA Methylation-Gold™ Kit (Zymo Research, Irvine, CA, USA) according to the manufacturer’s instructions. PCR was performed using 1 µL of bisulphite-converted DNA with a PyroMark PCR kit (Qiagen) according to the manufacturer’s instructions, with minor modifications. Methylation levels were quantified using PyroMark Q48 Autoprep and PyroMark software. Primer sequences and PCR conditions are shown in Supplementary Table S6.

### 2.5. Statistical Analyses

For statistical analysis of the distribution of 5-HTTLPR (/rs25531/rs25532) genotypes, the Chi-square test was applied. Nonparametric tests (Mann–Whitney U test or Kruskal–Wallis test) were applied to test for difference in continuous variables between two or more categorical variables. The Dunn test with adjustment for multiple comparisons was used as a post hoc pairwise test after significant results in the Kruskal–Wallis test, to determine which categorical variables differed from each other. Linear regression was used to assess whether one continuous variable had an impact on another continuous variable. 

To avoid type I errors, Bonferroni correction was applied to the *p*-values where multiple testing was conducted. As eight CpG-sites together with the mean value of all sites were considered in each methylation test, a *p*-value of 0.05/9 = 0.0056 was taken as threshold for statistical significance. Statistical results of methylation analyses were only reported for the mean of all CpG-sites.

All statistical analyses were performed in either SPSS (IBM, Armonk, NY, USA) or R (http://www.r-project.org). Figures were generated using R and RStudio [49], using the packages *ggplot2* and *ggpubr* [50,51].

## 3. Results

### 3.1. Genotyping

To investigate whether there was an association between *SLC6A4* promoter variants and GTS, we genotyped all the GTS individuals and the controls, and there was no statistically significant difference neither in the genotype distribution (*n* = 159, *p* = 0.243) nor in the allele frequencies of the 5-HTTLPR polymorphism (*n* = 318, *p* = 0.177) (Table 1). 

To determine the distribution of 5-HTTLPR/rs25531/rs25532 haplotypes (L_AC_ compared to all other combinations), we genotyped the two 5-HTTLPR-adjacent SNPs rs25531 (A>G) and rs25532 (C>T) in 41 GTS individuals and 40 controls homozygous for either the S or the L allele. We did not observe any significant difference in the haplotype distribution (*n* = 162, *p* = 0.538) in GTS individuals compared to controls (Table 1). As the rs25532 T allele was not observed in the L_G_ background, the L_GC_ haplotype will be referred to as L_G_, which is in line with the nomenclature from previous publications [17,38]. Finally, we investigated all the GTS individuals and 51 controls for the Ile425Val variant, which was not present in anyone.

### 3.2. Expression Analysis

**Figure 1:**
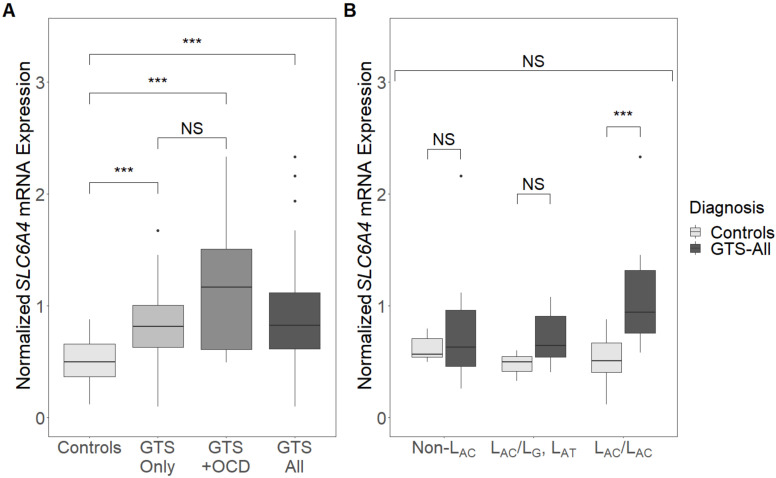
Expression levels of *SLC6A4* normalized to *GUSB* expression levels in (**A**) Gilles de la Tourette syndrome (GTS)-only, GTS+obsessive-compulsive disorder (OCD) or GTS—all individuals and controls and (**B**) GTS—all individuals and controls with different three-locus genotypes (L_AC_/L_AC_, L_AC_/L_G_ or L_AT_, or Non-L_AC_). Box plots indicate median, quartiles and outliers. ***, *p* < 0.001; NS, not significant.

We analysed *SLC6A4* mRNA expression levels using RT-qPCR in 57 GTS individuals (43 GTS-only and 14 GTS+OCD) and 36 controls from whom RNA was available and observed a significant difference (*n* = 93, *p* < 0.001) (Figure 1A). A three-way analysis between GTS-only, GTS+OCD and control individuals followed by a pairwise comparison showed significantly higher expression levels in both GTS-only and GTS+OCD individuals compared to controls ((*n* = 79, p.adj < 0.001) and (*n* = 50, p.adj < 0.001), respectively), while there was no significant difference between GTS-only and GTS+OCD individuals (*n* = 57, p.adj = 0.368) (Figure 1A).

To assess whether *SLC6A4* variants had a modifying effect on gene expression, we examined *SLC6A4* expression levels in GTS individuals and controls with regards to their 5-HTTLPR genotype and three-locus genotype (L_AC_/L_AC_, L_AC_/L_G_ or L_AT_, or non-L_AC_) and did not detect any statistically significant difference (GTS: *n* = 57, *p* = 0.555; controls: *n* = 36, *p* = 0.162) (Supplementary Figure S3). *SLC6A4* expression levels were, however, significantly higher in GTS individuals than in controls when only individuals with the L_AC_/L_AC_ three-locus genotype were considered (*n* = 27, *p* < 0.001) (Figure 1B). A difference in expression levels between GTS individuals and controls was not observed for the other genotypes (L_AC_/L_G_ or L_AT_: (*n* = 9, *p* = 0.167); Non-L_AC_: (*n* = 16, *p* = 1)). 

### 3.3. Methylation Analyses

To investigate whether the observed differences in *SLC6A4* expression could be associated with epigenetic regulation, we assessed the DNA methylation levels of eight CpG-sites at the promoter region of *SLC6A4* in all the GTS individuals (50 GTS-only and 22 GTS+OCD) and the controls. One individual (GTS+OCD) was omitted from the analysis, as the DNA sample repeatedly failed the pyrosequencing quality control. The eight CpG sites assessed in this study were selected from a region of the *SLC6A4* CpG island previously investigated in individuals with major depressive disorder [42] and ADHD [44].

There was no significant difference in the mean DNA methylation levels between GTS-only, GTS+OCD and controls (*n* = 158, *p* = 0.725, Supplementary Figure S4), and we did not observe any association between the mean methylation levels and the *SLC6A4* expression (*n* = 92, *p* = 0.992) or the 5-HTTLPR genotype (*n* = 158, *p* = 0.253). Furthermore, methylation levels were not associated with GTS when considering individuals with the L_AC_/L_AC_ three-locus genotype (*n* = 38, *p* = 0.884) nor any of the other three-locus genotypes (L_AC_/L_G_ or L_AT_: (*n* = 11, *p* = 0.850); Non-L_AC_: (*n* = 31, *p* = 0.842)).

## 4. Discussion

To investigate the involvement of the serotonin transporter SERT in GTS pathology, we performed expression analysis, genotyping and methylation analysis of *SLC6A4* in GTS individuals with and without OCD compared to healthy controls. We observed significantly higher *SLC6A4* mRNA levels in GTS individuals compared to controls (*p* < 0.001), with a tendency of higher expression levels of *SLC6A4* mRNA in GTS+OCD individuals compared to GTS-only, although this difference was not statistically significant. Elevated expression levels of *SLC6A4* were previously observed in GTS rat models [36], and taken together these results suggest that increased serotonin clearance due to overexpression of *SLC6A4* may contribute to GTS aetiology. The tendency of higher *SLC6A4* expression levels in GTS+OCD individuals than GTS-only may explain why selective serotonin reuptake inhibitors (SSRIs) are more effective in the treatment of OCD symptoms than the treatment of tics [52]. SSRIs increase the level of serotonin in the synaptic cleft by inhibiting reuptake of serotonin to the presynaptic neuron. If GTS+OCD individuals indeed do have a higher *SLC6A4* expression than those with only GTS, the use of SSRIs would also have a larger counteracting effect on the hyposerotonergic state resulting from the increased *SLC6A4* expression. These results are also in line with the recent study by Müller-Vahl andcolleagues, who have shown increased SERT BP in TS+OCD individuals, but not in TS-only individuals, and a significant overall reduction in SERT binding following SSRI treatment [34].

When only considering the individuals with the same genotype, higher expression levels were only observed in GTS individuals with the L_AC_/L_AC_ genotype compared to the controls. In a previous study, overall higher *SLC6A4* expression levels were detected in human and rat cell lines with the L_AC_/L_AC_ genotype when using reporter constructs [38], while in our study, the increased expression levels were only observed in GTS individuals, but not in the control individuals. This indicates that an overexpression of *SLC6A4* in the presence of the L_AC_/L_AC_ genotype is more protruding in GTS individuals with or without OCD, whereas the different three-locus genotypes do not seem to affect *SLC6A4* expression in healthy controls. 

In line with some of the other studies [16,53], we did not find any association between GTS diagnosis (with or without OCD) and *SLC6A4* 5-HTTLPR genotype nor allele distribution. Previously, the 5-HTTLPR/rs25331/rs25332 L_AC_ haplotype was associated with OCD [38], and it was shown to be more frequent in GTS individuals compared to controls, in particular in GTS individuals without OCD [17]. In this study, we did not find an association between GTS diagnosis and any of the 5-HTTLPR/rs25331/rs25332 haplotype variants. This is likely to be due to the small size of the present cohort, especially with regards to the number of GTS+OCD individuals (*n* = 14) included in the haplotype analysis. The third locus, rs25532, was included only in a few studies with OCD and/or GTS individuals [17,38,54], but in several other studies, only the distribution of the 5-HTTLPR or 5-HTTLPR/rs25531 variants were investigated [39,53,55,56]. This challenges comparison across studies, which will either be limited by the number of studies, or is potentially erroneous due to different genotyping methodologies. Further studies investigating the distribution and the effect of the three-locus haplotype in larger cohorts of GTS individuals with or without OCD are warranted to provide a clearer picture.

Promoter methylation of *SLC6A4* has not been investigated previously in individuals with GTS. In the present study, we did not observe any differences in the mean DNA methylation levels between GTS individuals with or without OCD and controls. We cannot, however, exclude that inclusion of further sites within the promoter CpG-island may affect the mean methylation levels, as we have investigated only eight selected CpG-sites, which were also employed in other studies [42,44]. In this study, differential methylation was investigated in blood, the only material available from the individuals. It is possible that brain regions, such as caudate nucleus tissue of the basal ganglia, the prefrontal cortex, the thalamus and the putamen known to be involved in GTS pathology, may be differentially methylated [57,58,59,60,61]. Another plausible explanation of the negative findings can be that serotonin expression in GTS individuals is not regulated by DNA methylation. 

The *SLC6A4* mRNA upregulation reported in the current study is furthermore in line with the dopamine hypothesis of GTS. Upregulation of SERT would result in increased serotonin clearance, and such a hyposerotonergic state is suggested to cause upregulation post-synaptic 5-HT_2A_ receptors, which may facilitate dopamine release [17,33]. At the same time, SERT is also capable of dopamine uptake [29]. An upregulation of *SLC6A4* expression could thus ultimately lead to abnormal levels of dopamine, a key component in GTS pathology. It is though warranted to investigate larger cohorts, as the size of the present cohort is relatively small. 

## 5. Conclusions

In this study, we show that *SLC6A4* expression is upregulated in GTS individuals, both with and without OCD, compared to controls. Although we did not observe any overrepresentation of any specific genotype in GTS individuals compared to controls, increased expression of *SLC6A4* in GTS individuals may be modulated by the L_AC_/L_AC_ genotype, as controls with this genotype had normal expression levels. DNA methylation levels at the promoter region of *SLC6A4* were not associated with the presence of GTS (with or without OCD), mRNA expression levels or individual genotypes, suggesting that *SLC6A4* expression is not regulated by DNA methylation of the investigated CpG-sites in the promotor region of the gene. 

## Figures and Tables

**Table 1:** Genotyping summary.

Variable		GTS Individuals	Controls	chi^2^	*p*-Value	OR
Genotype: no. (%)						
Total number of individuals		72	87			
	L/L	28 (38.9)	23 (26.4)			
	S/L	31 (43.1)	46 (52.9)			
	S/S	13 (18.1)	18 (20.7)	2.829	0.243	
Allele: no. (%)						
	L	87 (60.4)	92 (52.9)			
	S	57 (39.6)	82 (47.1)	1.822	0.177	1.360
Haplotype: no. (%)						
Total number of individuals		41	40			
	L_AC_	47 (57)	42 (52.5)			
	L_AT_, L_G_, S	35 (43)	38 (47.5)	0.380	0.538	1.2150

L, long allele; S, short allele; A and G, A- or G-allele in rs25531; T and C, T- or C-allele in rs25532; OR, odds ratio; chi^2^, chi-square.

## Data Availability

The data presented in this study are available in the Supplementary Materials.

## Supplementary Materials

The following are available online at https://www.mdpi.com/2073-4425/12/1/86/s1: Table S1—Patients and controls, Table S2—Genotyping *SLC6A4* (Ile425Val)—Primers and PCR conditions, Table S3—Genotyping *SLC6A4* (5-HTTLPR)—Primers and PCR condition, Table S4—cDNA synthesis, Table S5—Quantitative PCR of SLC6A4 and GUSB—Probes and qPCR conditions, Table S6—Methylation analysis—Primers and PCR conditions, Figure S1—Genomic location of 5-HTTLPR, rs25531 and rs25532, Figure S2—Genomic location of CpG-sites, Figure S3—Expression levels and 5-HTTLPR genotypes, Figure S4—Mean *SLC6A4* methylation levels.
