# Supplementary material for: Elevated Expression of SLC6A4 Encoding the Serotonin Transporter (SERT) in Gilles de la Tourette Syndrome
Source: Genes (Basel). 2021 Jan 12;12(1):86. doi: 10.3390/genes12010086 (PMC7827645; doi:10.3390/genes12010086)
Supplement: Supplementary file 1 [file genes-12-00086-s001.zip › Table S2.pdf]

**Gene**	**Primer**	**Sequence**
SLC6A4 (I425V)	Seq-tail:PCR FW	5’-**acccactgcttactggcttatc:**TGGAAGCCCCACCCTTCCTG-3’
	Seq-tail:PCR RV	5’-**gaggggcaaacaacagatggc:**CATCCTCCCACAGCCCATTTCC-3’
	**PCR Master mix (30 µl pr. reaction)**	
SLC6A4 (I425V)	100 ng DNA	
	0.5 µl of each primer (10 µM)	
	0.5 µl dNTP mix (2.5 mM of each)	
	6 µl 5x Q-solution	
	3 µl 10x PCR buffer	
	0.1 µl HotStarTaq® DNA polymerase	
	14.4 µl sterile H_2O	
**PCR conditions**		
95°C	15m	
95°C	1m	x40 cycles
65°C	1m	
72°C	1m	
72°C	7m	
	**Sequencing Master mix (pr. reaction)**	
SLC6A4 (I425V)	1-2 µl PCR product	
	1 µl BigDye™ Terminator v3.1 Cycle Sequencing	
	1.5 µl BigDye™ Terminator 5x Sequencing buffer	
	0.3 µl FW/RV sequencing primer	
	5.2-6.2 µl sterile H_2O	
**Sequencing conditions**		
98°C	1m	
98°C	10s	X25 cycles
50°C	5s	
60°C	4m	
