# Supplementary material for: Elevated Expression of SLC6A4 Encoding the Serotonin Transporter (SERT) in Gilles de la Tourette Syndrome
Source: Genes (Basel). 2021 Jan 12;12(1):86. doi: 10.3390/genes12010086 (PMC7827645; doi:10.3390/genes12010086)
Supplement: Supplementary file 1 [file genes-12-00086-s001.zip › Table S3.pdf]

| Gene | Primer | Sequence |
|---|---|---|
| *SLC6A4* (5-HTTLPR) | Seq-tail:PCR FW | 5'-**acccactgcttactggcttatc:**GGCGTTGCCGCTCTGAATGC-3' |
| | Seq-tail:PCR RV | 5'-**gaggggcaaacaacagatggc:**GAGGGACTGAGCTGGACAACCAC-3' |
| | **PCR Master mix (30 µl pr. reaction)** | |
| *SLC6A4* (5-HTTLPR) | 75 ng DNA | |
| | 2.625 µl of each primer (10 µM) | |
| | 2.6 µl dNTP mix (2.5 mM of each) | |
| | 6 µl 5x Q-solution | |
| | 3 µl 10x PCR buffer | |
| | 0.15 µl HotStarTaq® DNA polymerase | |
| | 11.7 µl sterile $H_2O$ | |
| **PCR conditions** | | |
| 95°C | 15m | |
| 95°C | 30s | X35 cycles |
| 68°C | 30s | |
| 72°C | 1m 30s | |
| 72°C | 10m | |
| | **Sequencing Master mix (pr. reaction)** | |
| *SLC6A4* (5-HTTLPR) | 2-3 µl PCR product | |
| | 1 µl BigDye™ Terminator v3.1 Cycle Sequencing | |
| | 1.5 µl BigDye™ Terminator 5x Sequencing buffer | |
| | 0.3 µl FW/RV sequencing primer | |
| | 4.2-5.2 µl sterile H2O | |
| **Sequencing conditions** | | |
| 98°C | 1m | |
| 98°C | 10s | X25 cycles |
| 50°C | 5s | |
| 60°C | 4m | |
