# Supplementary material for: Elevated Expression of SLC6A4 Encoding the Serotonin Transporter (SERT) in Gilles de la Tourette Syndrome
Source: Genes (Basel). 2021 Jan 12;12(1):86. doi: 10.3390/genes12010086 (PMC7827645; doi:10.3390/genes12010086)
Supplement: Supplementary file 1 [file genes-12-00086-s001.zip › Table S4.pdf]

cDNA Synthesis Master mix	
X µl sterile H_2O, depending on [RNA], to make final volume 20 µl.	
5 µl 10X RT buffer	
2 µl 25X dNTP Mix (100 mM)	
5 µl 10X Random Primer	
2.5 µl Multiscribe RT 50U/µl	
1 µg total RNA	
**Thermocycling conditions**	
25°C	10m
37°C	120m
85°C	5m
