# Supplementary material for: Elevated Expression of SLC6A4 Encoding the Serotonin Transporter (SERT) in Gilles de la Tourette Syndrome
Source: Genes (Basel). 2021 Jan 12;12(1):86. doi: 10.3390/genes12010086 (PMC7827645; doi:10.3390/genes12010086)
Supplement: Supplementary file 1 [file genes-12-00086-s001.zip › Table S5.pdf]

Gene	Probe	
SLC6A4	#Hs00169010_m1 (Applied Biosystems)	
GUSB	#Hs00939627_m1 (Applied Biosystems)	
**qPCR Master mix (20 µl pr. reaction)**		
9 µl cDNA (1.79 ng/ul)		
10 µl 2x TaqMan Universal PCR Master mix (Applied Biosystems)		
1 µl TaqMan probe (Applied Biosystems)		
**qPCR conditions**		
50°C	2m	
95°C	10m	
95°C	15s	X40 cycles
60°C	1m	
