# Supplementary material for: Elevated Expression of SLC6A4 Encoding the Serotonin Transporter (SERT) in Gilles de la Tourette Syndrome
Source: Genes (Basel). 2021 Jan 12;12(1):86. doi: 10.3390/genes12010086 (PMC7827645; doi:10.3390/genes12010086)
Supplement: Supplementary file 1 [file genes-12-00086-s001.zip › Table S6.pdf]

Gene	Primer	Sequence
SLC6A4	PCR F	GTTTTAGGAAGAAAGAGAGAGTAGTT
	PCR R	[BIO]ATCCTAACTTTCCTACTCTTTAACT
	Seq 1	GGAAGATGGGGAGGTGTT
	Seq 2	GGTAAGAGTAGATTTTTGTGTG
	**PCR Modifications**	
SLC6A4	0.15µM of each primer	
	1µl $MgCl_2$ Per reaction	
**PCR conditions**		
95°C	15m	
94°C	30s	x45 cycles
56°C	30s	
72°C	30s	
72°C	10m	
