# Supplementary material for: Elevated Expression of SLC6A4 Encoding the Serotonin Transporter (SERT) in Gilles de la Tourette Syndrome
Source: Genes (Basel). 2021 Jan 12;12(1):86. doi: 10.3390/genes12010086 (PMC7827645; doi:10.3390/genes12010086)
Supplement: Supplementary file 1 [file genes-12-00086-s001.zip › Figure S2.pdf]

CGGGATGGGGACGATGGGGAGGTGTC**CG**AGGTCAAGAGAAAG**CG**GCA**CG**A
GCAGACCCCTGTGTGC**CG**TCCTGTGGG**CGCG**GGG**CG**GCAGGGGAGG**CG**CA
CACCTGCTCCTTTGTGCAGCCTCCCCCCTCCCGCAAAGTTAAAGAGCAGG
AAAGTCAGGATTCCTCGCTCGGCCCTGCCCTGCCGGCTGCTCCGCGCTCC
GCTCCTCCCTGCGAGCGTGTGTGTGTGTCGGGGGTCCCTCCCCTCCTGGC
TCTGGGGTCGGGCGCGCACCCCGCCCCGTAGCGCGGCCCCTCCCTGGCGA
GCGCAACCCCATCCAGCGGGAGCGCGGAGCCGCGGCCGCGGGGAAGCATT
AAGTTTATTCGCCTCAAAGTGACGCAAAAATTCTTCAAGAGCTCTTTGGC
GGCGGCTATCTAGAGATCAGACCATGTGAGGGCCCGCGGGTACAAATACG
GCCGCGCCGGCGCCCCTCCGCACAGCCAGCGCCGCCGGGTGCCTCGAGGG
CGCGAGGCCAGCCCGCCTGCCCAGCCCGGGACCAGCCTCCCCGCGCAGCC
TGGCAGGTGGGTCCGCTTTTCCTCTCCGCCTCGAACCCACGTTTCTTTCC
AGACCTTCTTCCCCGCCTCGGGGAGGGGGATAGAACCGCTGCGCCCCACC
GCCCTGCGAGGAGGCGAGGAGGTGCATGCGCCCCAGCGGTGGGCGCCGGA
TCCTGCCCCTGCGCCCTCCACGCTCAGCAAGAGCCAGAGCTGAAGCTGAC
CGGCCAGAGTGGGAGACGAGGAACGTGGAGTGCTCGAAGTGGGCGGGCG
