# Supplementary material for: Elevated Expression of SLC6A4 Encoding the Serotonin Transporter (SERT) in Gilles de la Tourette Syndrome
Source: Genes (Basel). 2021 Jan 12;12(1):86. doi: 10.3390/genes12010086 (PMC7827645; doi:10.3390/genes12010086)
Supplement: Supplementary file 1 [file genes-12-00086-s001.zip › Supplementary material - tables and figures text.docx]

Supplementary Material

**Table S1**: Overview of patient and control cohort

Genotyping of 5-HTTLPR/rs25531/rs25532 was only carried out on individuals homozygous for the 5-HTTLPR variant (i.e. individuals with the SS- or LL-genotype). 5-HTTLPR/rs25531/rs25532 expression analysis was only carried out on individuals homozygous for the 5-HTTLPR variant from whom we had obtained RNA.

**Table S2**: Genotyping *SLC6A4* (I425V) - Primers and PCR conditions

**Table S3**: Genotyping *SLC6A4* (5-HTTLPR) - Primers and PCR conditions

**Table S4**: cDNA synthesis

**Table S5**: Quantitative PCR of *SLC6A4* and *GUSB* – Probes and qPCR conditions

**Table S6**: Methylation analysis - Primers and PCR conditions

**Figure S1**: Genomic location of 5-HTTLPR, rs25531 and rs25532 (Position: chr17:28563985-28564513). The amplicon sequence (reverse complemented) including forward and reverse primers highlighted in yellow and the 43 bp insertion shown highlighted in red. The SNP rs25531 (A/G) is highlighted in green and rs25532 (C/T) highlighted in blue.

**Figure S2**: Genomic location of CpG-sites. The full sequence (reverse complemented) of the CpG-island (Position: [chr17:28562388-28563186](https://genome-euro.ucsc.edu/cgi-bin/hgTracks?hgsid=232695817_RHZdp9ZnDxHA9iqAvTbJ5lQslYgQ&db=hg19&position=chr17%3A28562388-28563186)) is shown, and CpG-sites are highlighted in grey. CpG-sites assayed in this study are in bold and underlined with red.

**Figure S3**: *SLC6A4* expression levels dependent on 5-HTTLPR genotype. Expression levels of SLC6A4 normalized to GUSB expression levels in GTS individuals and controls with different genotypes. L, long allele; S, short allele. Box plots indicate median, quartiles and outliers.

**Figure S4**: Mean *SLC6A4* methylation levels of all 8 CpG sites. Box plots indicate median, quartiles and outliers.
