# Supplementary material for: Elevated Expression of SLC6A4 Encoding the Serotonin Transporter (SERT) in Gilles de la Tourette Syndrome
Source: Genes (Basel). 2021 Jan 12;12(1):86. doi: 10.3390/genes12010086 (PMC7827645; doi:10.3390/genes12010086)
Supplement: Supplementary file 1 [file genes-12-00086-s001.zip › Table S1.pdf]

Analysis	GTS individuals		Controls
	GTS-Only	GTS+OCD	
**Total**	50	22	87
**Genotyping**			
5-HTTLPR	50	22	87
5-HTTLPR/rs25531/rs25532	27	14	40
Ile425Val	50	22	51
**Expression analyses**			
SLC6A4	43	14	36
5-HTTLPR/rs25531/rs25532	23	9	20
**Methylation analysis**			
SLC6A4	50	21	87
