# Supplementary figures and images for: Elevated Expression of SLC6A4 Encoding the Serotonin Transporter (SERT) in Gilles de la Tourette Syndrome
Source: Genes (Basel). 2021 Jan 12;12(1):86. doi: 10.3390/genes12010086 (PMC7827645; doi:10.3390/genes12010086)

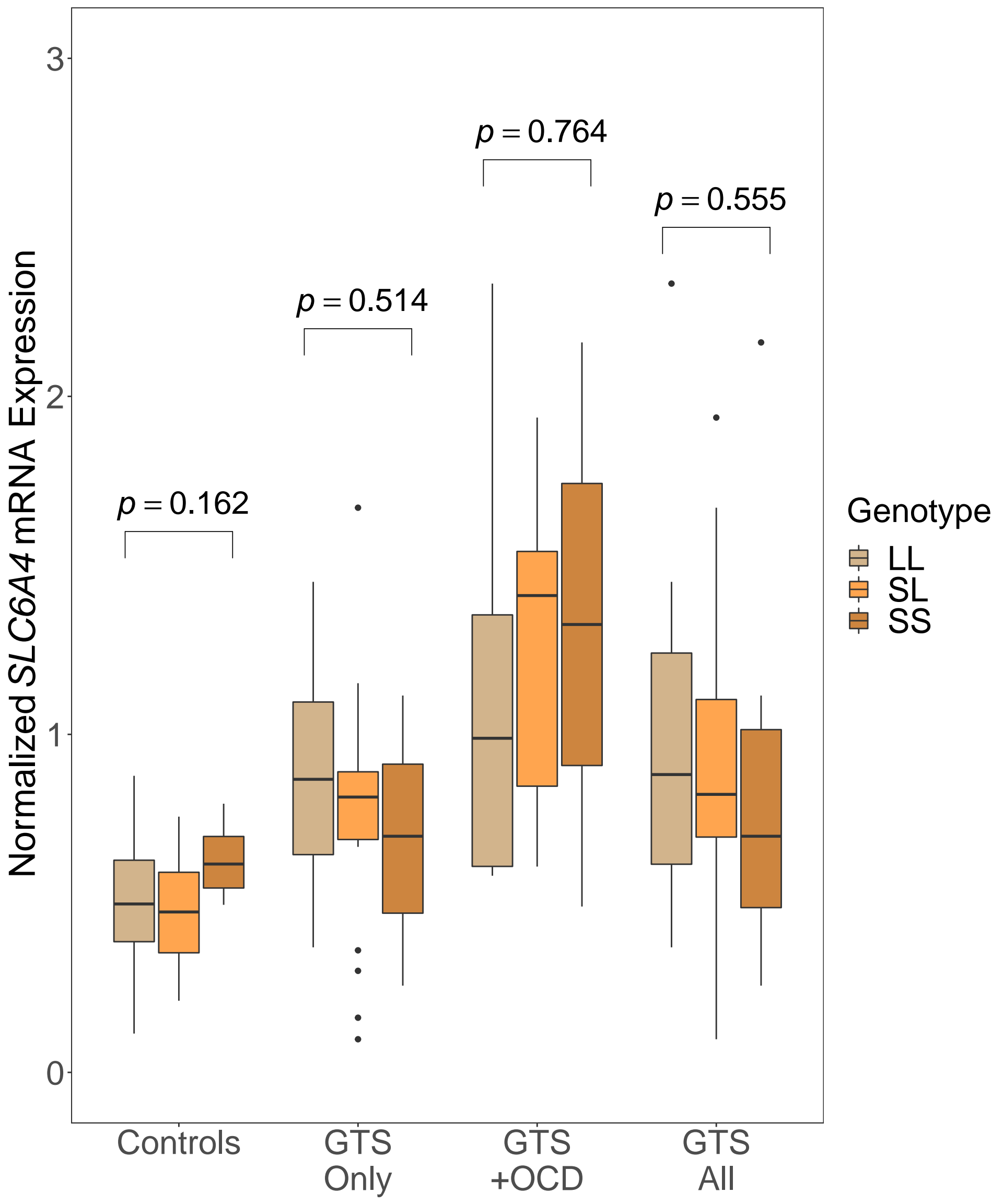

Supplement: Supplementary file 1 [file genes-12-00086-s001.zip › Figure S3.pdf]

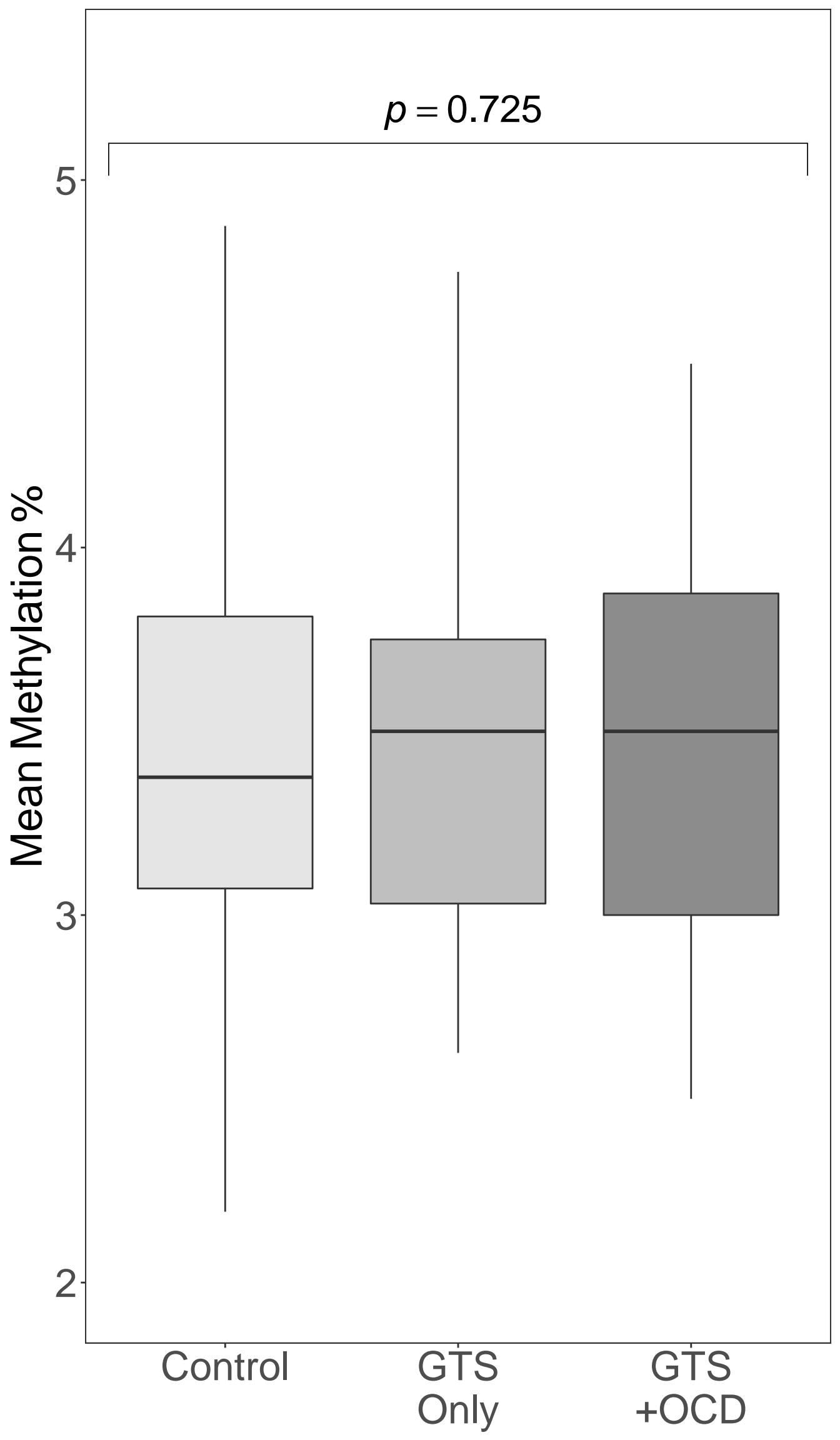

Supplement: Supplementary file 1 [file genes-12-00086-s001.zip › Figure S4.pdf]
